# Is Hypoxia a Factor Influencing PSMA-Directed Radioligand Therapy?—An In Silico Study on the Role of Chronic Hypoxia in Prostate Cancer

**DOI:** 10.3390/cancers13143429

**Published:** 2021-07-08

**Authors:** Gabriele Birindelli, Milos Drobnjakovic, Volker Morath, Katja Steiger, Calogero D’Alessandria, Eleni Gourni, Ali Afshar-Oromieh, Wolfgang Weber, Axel Rominger, Matthias Eiber, Kuangyu Shi

**Affiliations:** 1 Department of Nuclear Medicine, Inselspital, University of Bern, 3010 Bern, Switzerland; milos.drobnjakovic@students.unibe.ch (M.D.); eleni.gourni@insel.ch (E.G.); ali.afshar@insel.ch (A.A.-O.); axel.rominger@insel.ch (A.R.); kuangyu.shi@dbmr.unibe.ch (K.S.); 2 Department of Nuclear Medicine, Klinikum Rechts der Isar, School of Medicine, Technical University of Munich, 81675 München, Germany; v.morath@tum.de (V.M.); calogero.dalessandria@tum.de (C.D.); w.weber@tum.de (W.W.); matthias.eiber@tum.de (M.E.); 3 Institute of Pathology, School of Medicine, Technical University of Munich, 81675 München, Germany; katja.steiger@tum.de

**Keywords:** hypoxia, radioligand therapy, tumor microenvironment, convection–reaction–diffusion models, dosimetry, radiobiology

## Abstract

**Simple Summary:**

Tumor hypoxia is considered a critical factor associated with the resistance of conventional radiotherapy, where the X-ray-induced free radicals lead to DNA damage in a manner that is strongly dependent on the tissue oxygenation. The emerging PSMA-directed radioligand therapy (RLT) employs the α or β particles emitted by the radiopharmaceuticals to kill the tumor cells. In contrast to conventional therapy, the induced DNA damage is less dependent on the oxygenation status. Less attention has been paid to investigating whether tumor hypoxia will influence the efficacy of PSMA-directed RLT. We propose a histology-driven in silico model to quantitatively investigate the influence of tumor hypoxia on the treatment outcome for PSMA-directed RLT with ${}^{177}$Lu and ${}^{225}$Ac. Our finding suggests that hypoxia is a factor to be considered for the application of PSMA-directed RLT.

**Abstract:**

Radioligand therapy (RLT) targeting prostate specific-membrane antigen (PSMA) is an emerging treatment for metastatic castration-resistant prostate cancer (mCRPC). It administrates ${}^{225}$Ac- or ${}^{177}$Lu-labeled ligands for the targeted killing of tumor cells. Differently from X- or γ-ray, for the emitted α or β particles the ionization of the DNA molecule is less dependent on the tissue oxygenation status. Furthermore, the diffusion range of electrons in a tumor is much larger than the volume typically spanned by hypoxic regions. Therefore, hypoxia is less investigated as an influential factor for PSMA-directed RLT, in particular with β emitters. This study proposes an in silico approach to theoretically investigate the influence of tumor hypoxia on the PSMA-directed RLT. Based on mice histology images, the distribution of the radiopharmaceuticals was simulated with an in silico PBPK-based convection–reaction–diffusion model. Three anti-CD31 immunohistochemistry slices were used to simulate the tumor microenvironment. Ten regions of interest with varying hypoxia severity were analyzed. A kernel-based method was developed for dose calculation. The cell survival probability was calculated according to the linear-quadratic model. The statistical analysis performed on all the regions of interest (ROIs) shows more heterogeneous dose distributions obtained with ${}^{225}$Ac compared to ${}^{177}$Lu. The higher homogeneity of ${}^{177}$Lu-PSMA-ligand treatment is due to the larger range covered by the emitted β particles. The dose-to-tissue histogram (DTH) metric shows that in poorly vascularized ROIs only 10% of radiobiological hypoxic tissue receives the target dose using ${}^{177}$Lu-PSMA-ligand treatment. This percentage drops down to 5% using ${}^{225}$Ac. In highly vascularized ROIs, the percentage of hypoxic tissue receiving the target dose increases to more than 85% and 65% for the ${}^{177}$Lu and ${}^{225}$Ac-PSMA-ligands, respectively. The in silico study demonstrated that the reduced vascularization of the tumor strongly influences the dose delivered by PSMA-directed RLT, especially in hypoxic regions and consequently the treatment outcome.

## 1. Introduction

Hypoxia is an oxygen deprivation state within a region of a tissue or an organ that induces a complex systemic and cellular response [1]. In tumors, hypoxic regions arise from abnormal tumor vessel structure and function, limiting perfusion to the tissue [2]. Chronic hypoxia is a quasi-steady state condition principally due to the spatial disorganization of tumor vessels that can create large inter-capillary distances surpassing the oxygen diffusion range [3]. Tumor hypoxia is considered as one of the main resistance factors for radiotherapy [4].

Radiation therapy (RT) is among the most important and common techniques that are adopted for cancer treatment. The aim of radiotherapy consists in killing cancer cells with the use of ionizing radiations that are able to spare the healthy tissue surrounding the tumor volume. Ionizing radiation primarily affects the cell by breaking covalent bonds within the genomic DNA. Strand breaks can be subdivided into single-strand breaks (SSB) and double-strand breaks (DSB), where opposite strands are damaged in close proximity. DNA double-strand breaks are widely accepted as the key driver of radiobiological effects in cells. While the yield of DSBs is linear over a dose range spanning from 1 mGy to 100 Gy [5], another important role is played by the type of particles used for the treatment. Indeed, heavy charged particles such as protons or α particles deposit their energy in a dramatically lower range with respect to photons or electrons. The quantity that defines the amount of energy delivered in a space unit is the linear energy transfer (LET). To a higher LET corresponds a denser distribution of ionizations in DNA and thus a greater level of biological damages [6,7]. A substantial secondary effect is the generation of reactive oxygen species (ROS) that hinder normal cellular functions by oxidizing proteins, lipids, and DNA. Cumulatively, the two different mechanisms cause cell lethality and mitotic failure. In a hypoxic environment, there is a lack of substrate for ROS generation [8]. The enhancing effect of oxygen on radiation-induced damage can be quantified by the oxygen-enhancement ratio (OER). The oxygen-enhancement ratio is defined as the ratio of the doses needed to produce a given level of injury in the absence and presence of oxygen. OER is particle dependent and it is an important factor to consider for the tumor control probability [9].

The last decades have witnessed rapid acceleration in the impact of tumor-targeted radioligand therapy (RLT). Promising results have been obtained in the treatment of metastatic castration-resistant prostate cancer (mCRPC), known for its high morbidity and mortality [10]. Prostate carcinoma is characterized by the increased expression of the specific type II transmembrane glycoprotein named prostate-specific membrane antigen (PSMA) [11]. Several studies have demonstrated the efficacy and safety of RLT for the treatment of mCRPC performed with Lutetium-177 (${}^{177}$Lu-PSMA-ligands) [12,13]. A recent meta-analysis shows biochemical and radiological responses in 46% and 37% of the patients, respectively [14]. Despite the early success, due to the relatively low linear energy transfer (LET) the β particle emitted by ${}^{177}$Lu causes mostly single-strained DNA breaks leading to treatment resistance. A more potent alternative is represented by the α-emitter Actinium-225 (${}^{225}$Ac-PSMA-ligands). The α particles emitted by ${}^{225}$Ac, with LET (∼100 keV/μm) 500 times higher compared to the β particle emitted by ${}^{177}$Lu, induces with a higher probability a double-strand break of the DNA [15,16]. A recent meta-analysis on ${}^{225}$Ac-PSMA-ligands therapy indicates a remarkable efficacy of this targeted approach, with a response rates that exceed those published for ${}^{177}$Lu-PSMA RLT [17]. Coherently with other solid tumors, hypoxic regions exist in prostate cancer, with a degree comparable to other malignancies [18,19,20,21]. Furthermore, hypoxia in prostate cancer has been demonstrated to correlate with increased tumor invasiveness, metastasis, and resistance to chemotherapy and radiotherapy [21]. In addition to factors common for all radiation-based treatments, the efficacy of radioligand therapy also depends on the ability of the radiopharmaceutical to reach and bind its target. The irregular spatial distribution of the vessels within the tumor makes certain regions difficult for the therapeutic to reach the cancer cells. While not directly a consequence of hypoxia, the lack of oxygen in a region is highly correlated to the inability of other molecules to reach it and could be considered an indirect effect on the treatment outcome [3]. Due to above-mentioned effects of hypoxia, a treatment plan should ideally consider the hypoxia severity of the tumor and optimize the treatment accordingly. Indeed, the reduced range of α particles in human tissues, i.e., ∼50 μm for α particles emitted by ${}^{225}$Ac with respect to ∼1.6 mm for β particles emitted by ${}^{177}$Lu, can strongly reduce the cross-fire effect in large tumors with poorly vascularized regions.

The complementary advantages of α and β-emitting RLTs lead to the concept of “cocktail treatment” to maximize the antitumoral effect. However, determining the local oxygen and drug distribution within a clinical setting is challenging and it remains unclear how such a cocktail treatment should be formulated and the potential treatment outcome. In the past decades, systems medicine has emerged as a tool to facilitate hypothesis generation, data integration, and patient-specific therapeutic development. Systems medicine has already been applied for the development of multi-modal imaging strategies [22]. Numerical investigations have been conducted to model the distribution of hypoxia in tumor microenvironment [23,24,25,26,27,28,29]. In these studies hypoxia is indirectly estimated by establishing the computational model on the microvascular distribution [23,24,25]. The results have been reported to be consistent with experimental measurements [26,27,28,29] and this strategy is often used in theoretical investigation of phenomena related to tumor hypoxia (e.g., imaging) [26,27,28,29]. However, the underlying assumption that hypoxia is induced by the limited oxygen diffusion in poorly vascularized tumor micro-areas constrains the microvasculature-based modeling mainly to chronic hypoxia [26,27,28,29]. In this work we propose an in silico modeling based on physiologically-based pharmaco-kinetic (PBPK) models to study the effect of chronic hypoxia on the treatment outcome in RLT for prostate cancer. The effect of chronic hypoxia is also examined in the context of radiobiological efficacy.

## 2. Materials and Methods

### 2.1. Animal Experiments

The PSMA-positive prostate carcinoma cell line LNCaP (purchased from the ATCC) was cultivated at 37 ${}^{\circ }$C in a humidified 5% CO${}_{2}$ atmosphere using RPMI 1640 medium supplemented with 10% (*v/v*) FBS and Pen-Strep [30,31]. Animal experiments were conducted with permission from the District Government of Upper Bavaria (application No.: 55.2-1-54-2532-216-15) according to the guidelines for the welfare and use of animals in cancer research. Male CB17-SCID mice at an age of 6 weeks were purchased from Charles River Laboratories (Wilmington, MA, USA) and held under specific pathogen free (SPF) conditions. Tumor inoculation was performed by mixing a cell suspension containing 5 × 10${}^{6}$ LNCaP cells 1:1 (*v/v*) with Matrigel (Corning, NY, USA) and subsequent subcutaneous injection above the shoulder. When tumors had reached ∼1 cm in size, the animals were sacrificed, xenograft tumors were excised and fixed in 10% (*v/v*) neutral-buffered formalin solution (Otto Fischar, Saarbrücken, Germany).

### 2.2. Stained Tumor Sections

The formalin-fixed, paraffin-embedded (FFPE) tissue was used to prepare consecutive 2 μm sections using a microtome (Microm). The immunohistochemistry (IHC) for CD31 (vessel endothelium) was performed with a primary rabbit anti-CD31 antibody (Abcam ab28364, 1:50) processed and detected on a Bond RXm system (Leica) with a polymer detection kit (without post primary antibody). The tissue was deparaffinized, pretreated with hydrogen-peroxide, incubated with the primary antibody for 15 min at room temperature, and the detection was performed with an anti-rabbit HRP polymer and DAB. Counterstaining was performed with hematoxylin. The slides were then dehydrated and coverslipped. Positive staining occurred as a dark-brown precipitate.

### 2.3. Computational Domain and Vessel Map Generation

**Figure 1:**
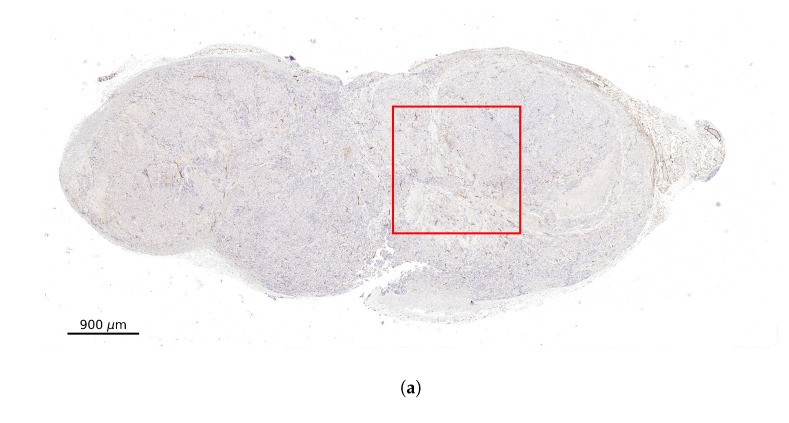

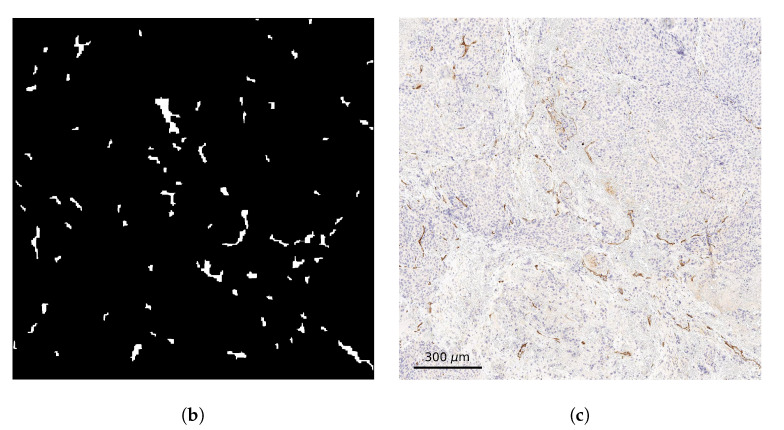
(**a**) Example of the used tissue microsection immunohistochemically stained for CD31 (vessel endothelium). The red square represents one of the regions of interest used for simulations. (**b**) Region of interest after binarization. (**c**) Zoom image of the ROI.

The computational domain is based on xenografts. The following steps were used for processing three different images of tissue slices that were immunohistochemically stained for vessel endothelium. As a first step, ten different regions of interest (ROIs) of 1.6×1.6 mm${}^{2}$ have been identified on the stained histology slices (Figure 1a). The ROIs have been selected in order to simulate the radiopharmaceuticals distribution in domains with different degrees of vascularisation. The selected ROIs have been binarized with the use of the automated Huang threshold method implemented in ImageJ (V.2.1.0) [32]. The threshold parameters have been adjusted in order to obtain the best vessels to background ratio. As a second step a specifically coded Python program has been used to clean the residual noise keeping only the vessels contours. The Python script can be downloaded by the reader (https://www.dropbox.com/sh/fy5ud6f7vovh4ty/AABPbDhbONdqus4OAJEFNUmGa?dl=0, accessed date 13 June 2021). The resulting image shown in Figure 1b is used as the computational domain. For each ROI a visual comparison of the obtained vessel contours with respect to the original image has been performed in order to assure the robustness of the algorithm. The vascular fraction of the domains spans between 1.0% and 3.2% with a mean value of 2.1%. The vascular fraction in the ROIs are comparable with other published values [33,34].

### 2.4. Multi-Scale Spatial-Temporal Models of PSMA-Ligands Dynamics

As shown in Section 2.3, the computational domain consists of extravascular space interspersed by vessels, which constitute holes in the mesh. The tumor tissue, i.e., the extravascular space, is assumed to be homogeneously divided into two sub-compartments: The interstitial compartment where the labelled PSMA-ligands can move and bind to the cells surface and the cellular compartment. The radiopharmaceutical enters the tumour interstitium from the vasculature. It is then transported through the interstitial volume by diffusion down concentration gradients and convection from regions of high to low interstitial fluid pressure (IFP). Finally, it exits via cellular uptake and backflow into the vasculature. The flux density of PSMA-ligands across vessel walls, $J_{v}$ (nmol s${}^{-1}$ cm${}^{-2}$), is assumed to be proportional to the difference between the concentrations on the vascular $C_{v}$ (nmol ml${}^{-1}$) and the interstitium $C_{i}$ side:

(1) $$J_{v}=L_{v}\left(C_{v}-C_{i}\right)$$

where $L_{v}$ (cm s${}^{-1}$) represents the vessel wall permeability to PSMA-ligands. The spatio-temporal evolution of the interstitial PSMA-ligands concentration can be described by a convection–reaction–diffusion (CRD) equation as:

(2) $$\partial_{t}C_{i}=\nabla \cdot \left(D_{\mathrm{PSMA}}\nabla C_{i}\right)-\nabla \cdot \left(\vec{v}\;R_{f}C_{i}\right)-k_{\mathrm{on}}C_{i}\left(R_{0}-C_{b}\right)+k_{\mathrm{off}}C_{b}-\lambda_{\mathrm{dec}}C_{i}$$

where $D_{\mathrm{PSMA}}$ is the diffusivity of PSMA-ligands, $R_{f}$ is the movement coefficient between the molecule and its carrier, the terms $\nabla \cdot (D_{\mathrm{PSMA}}\nabla C_{i})$ and $\nabla \cdot (\vec{v}\;R_{f}C_{i})$ describe changes in interstitial radiopharmaceutical concentration due to diffusion and convection, $k_{\mathrm{on}}$ (cm${}^{3}$ nmol${}^{-1}$ s${}^{-1}$) and $k_{\mathrm{off}}$ (s${}^{-1}$) are, respectively, the association and dissociation rates, $C_{b}$ is the bounded radiopharmaceutical concentration (nmol ml${}^{-1}$), $R_{0}$ (nmol ml${}^{-1}$) is the PSMA binding sites density and $\lambda_{\mathrm{dec}}$ (s${}^{-1}$) is the radionuclide decay constant. The labelled molecule has a high-binding affinity for PSMA expressed on the prostate cancer cell surface. Moreover, PSMA undergoes an internalisation process that allows the radionuclide to be concentrated within the cell [35]. Demanding mass conservation and assuming first-order kinetics, the rates of change of bounded and internalized PSMA-ligands concentrations, $C_{\mathrm{int}}$, can be written as

(3) $$\begin{array}{cc} \partial_{t}C_{b} & =k_{\mathrm{on}}C_{i}\left(R_{0}-C_{b}\right)-k_{\mathrm{off}}C_{b}-k_{\mathrm{int}}C_{b}-\lambda_{\mathrm{dec}}C_{b}, \end{array}$$

(4) $$\begin{array}{cc} \partial_{t}C_{\mathrm{int}} & =k_{\mathrm{int}}C_{b}FV_{i}/FV_{c}-k_{\mathrm{rel}}C_{\mathrm{int}}-\lambda_{\mathrm{dec}}C_{\mathrm{int}}, \end{array}$$

where $k_{\mathrm{int}}$ (s${}^{-1}$) and $k_{\mathrm{rel}}$ (s${}^{-1}$) are the internalization and the release rates, respectively, and $FV_{i}$ and $FV_{c}$ are the fractional volumes of tumour interstitium and cells within a voxel, respectively. The radiopharmaceutical flux across the vessel walls is modelled via Neumann boundary conditions imposed on the vessel boundaries as

(5) $$\vec{n}\cdot \left(D_{\mathrm{PSMA}}\nabla C_{i}\right)=J_{v}$$

where $\vec{n}$ is the normal unit vector to the respective boundary segment. No-flux boundary conditions were applied to the edges of the vessel map. Table 1 lists the parameter estimates deduced from the literature [36,37,38]. The ${}^{177}$Lu-PSMA-ligands and ${}^{225}$Ac-PSMA-ligands distributions in tumor microenvironment have been calculated by numerically solving the model presented above with a finite element method (FEM) implemented in FreeFem++ [39].

### 2.5. Tissue Oxygenation Models and Oxygen Dependent Tissue Segmentation

To study the effect of the presence of hypoxic tissue on the treatment outcome, we simulated the tissue oxygenation. Accordingly to the published literature, the oxygen transport is modelled as a purely diffusive process that is in equilibrium with cellular oxygen consumption [25,26,27,29]. The cellular oxygen consumption has been modelled considering Michaelis–Menten kinetics [25]. The reaction-diffusion equation for tissue oxygenation reads:

(6) $$\partial_{t}P=\nabla \cdot \left(D_{O_{2}}\nabla P\right)-\frac{M_{0}P}{P+P_{0}}$$

where *P* (mmHg) is the oxygen tension, $D_{O_{2}}$ (cm${}^{2}$ s${}^{-1}$) is the diffusion constant, $M_{0}$ (mmHg s${}^{-1}$) is the consumption rate at maximum oxygen levels and $P_{0}$ (mmHg) is the Michaelis-Menten coefficient of oxygen consumption. Accordingly to Mönnich et al. [27], the oxygen flux density across the vessel walls is considered to be proportional to the difference between the oxygen tension in erythrocytes $P_{\mathrm{ie}}$ (mmHg) and on the extravascular side of the wall *P*:

(7) $$J_{O_{2}}=L_{O_{2}}\left(P_{\mathrm{ie}}-P\right).$$

Similarly the model presented in Section 2.4, the oxygen flux across the vessel walls is modelled via Neumann boundary conditions imposed on the vessel frontiers as

(8) $$\vec{n}\cdot \left(D_{O_{2}}\nabla P\right)=J_{O_{2}}.$$

No-flux boundary conditions were applied to the edges of the vessel map. The parameters used in the oxygenation reaction-diffusion model have been derived by literature [27,29] and are listed in Table 2. The quasi-static oxygen distribution in the tumor microenvironment has been calculated by numerically solving Equation (6) with a FEM implemented in FreeFem++ [39]. The stationary approximation is reached by imposing a threshold of 0.1% (s${}^{-1}$) to the temporal variations of the solution.

**Figure 2:**
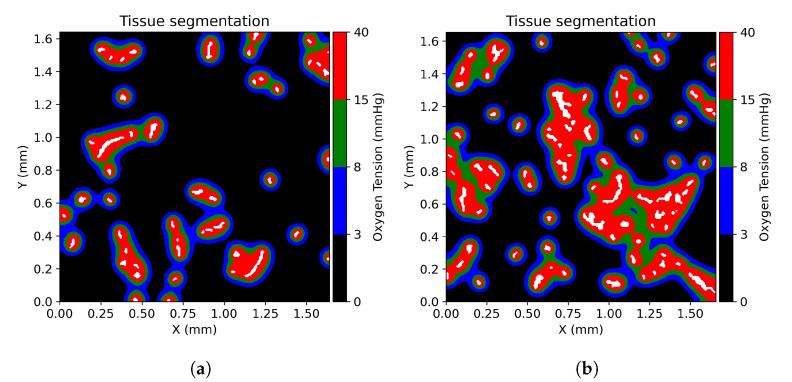

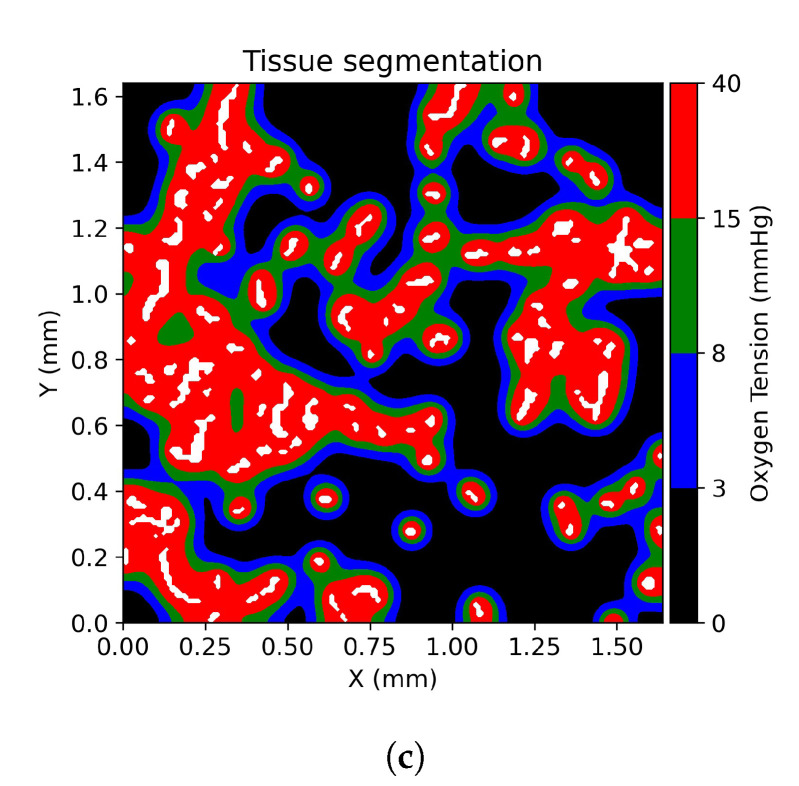
Tissue segmentation of the three representative ROIs based on the oxygenation level. Physoxia is labeled with red color, physiological hypoxia is labeled with green color, pathological hypoxia is labeled with blue color, and radiobiological hypoxia is labeled with black color. (**a**) Tissue segmentation for ROI A with a vascular fraction of 1%. (**b**) Tissue segmentation for ROI B with a vascular fraction of 2.34%. (**c**) Tissue segmentation for ROI C with a vascular fraction of 3.2%.

According to Mckeown [40], we defined physoxia (the oxygen tension between 40 mmHg and 15 mmHg), physiological hypoxia (the oxygen tension between 15 mmHg and 8 mmHg), pathological hypoxia (the oxygen tension between 8 mmHg and 3 mmHg), and radiobiological hypoxia (the oxygen tension below 3 mmHg). The extravascular matrix has been segmented into the previously defined regions accordingly to the solution of Equation (6) as shown in Figure 2.

### 2.6. Physiologically Based Pharmacokinetic Model

Physiologically based pharmacokinetic (PBPK) models are compartment-based models used to simulate the absorption, distribution, metabolism, and excretion of the injected compound in the human body [41]. An existing and validated PBPK model [38] for RLT studies has been adopted to calculate the arterial input function (AIF), i.e., the time dependant vascular concentration of PSMA-ligands $C_{v}$, used in our CRD model. The PBPK model for PSMA-ligands was adapted to be compatible with mouse physiology. Mouse physiological variables were either based on rescaling human parameters or derived from literature [42,43,44,45,46]. The tumor tissue was treated as a xenograft; therefore, parameters such as vascular and interstitial fraction, receptor density, PSMA-ligands binding constants, degradation and internalization rates were assumed to be the same as in humans [38]. The injected amount of radiopharmaceutical was chosen to obtain a mean deposited dose in the prostate tumor model of 10 Gy twenty days post-injection.

### 2.7. Absorbed Dose and Cell Survival Probability

The absorbed dose in each voxel of the domain has been calculated accordingly to the MIRD formalism [47]. The mean absorbed dose $D(r_{T},T_{D})$ (Gy) to target voxel $r_{T}$ over a defined period $T_{D}$ is defined as

(9) $$D\left(r_{T},T_{D}\right)=\sum_{r_{s}}\tilde{A}\left(r_{S},T_{D}\right)S\left(r_{T}\leftarrow r_{S}\right)$$

where $\tilde{A}\left(r_{S},T_{D}\right)$ (Bq) is the time-integrated activity in source voxel $r_{S}$ over the period $T_{D}$ and $S(r_{T}\leftarrow r_{S})$ is the radionuclide-specific quantity representing the mean absorbed dose to the target voxel $r_{T}$ per unit activity present in the source voxel $r_{S}$. In this work the *S* values for each radionuclide are calculated with the use of MIRD-cell (V2.0) [48]. The probability that a cell survives is calculated accordingly to the linear quadratic model

(10) $$P_{S}=e^{-\alpha D-\beta D^{2}}$$

where α and β are the linear quadratic parameters that characterize the cellular response to the ionizing radiation and *D* (Gy) is the absorbed dose [49]. The linear quadratic parameters depend on the tissue oxygenation: The higher is the oxygenation, the more radiosensitive are the cells. In this work, the oxygen level within a pixel is used to fit the linear quadratic parameters. The cell survival curve for α particle radiation is log-linear at low as well as high absorbed doses. Equation (10) can be consequently simplified as

(11) $$P_{S}=e^{-D/D_{0}}$$

where $D_{0}$ is the absorbed dose required to yield a survival fraction of 37% [50]. The parameters used to calculate the cells survival probability are resumed in Table 3.

## 3. Results

### 3.1. Tissue Oxygenation and Oxygen Dependent Tissue Segmentation

As a benchmark on the consistency of the oxygenation simulations with respect to the existing literature, for each ROI the median pO${}_{2}$ has been calculated. The resulting pO${}_{2}$ median calculated for each ROI spans in the range between 0.5 mmHg and 7.65 mmHg depending on the vascularization degree of the domain. These values are in agreement with the reported literature values for prostate tumors [40]. The main characteristic of the simulated oxygen distribution is the sharp gradient in the proximity of vessels and an asymptotic behavior approaching zero at large distances, which is consistent with previous investigations [25,27,29]. Consequently, the vascular fraction and the vessel distribution have a strong influence on the spatial distribution of each of the defined oxygen levels. Figure 2 shows the classification of the microenvironment tissue in three different ROIs, according to the definition given by Mckeown [40]. These ROIs have been selected as representative cases for a poorly vascularized tumor microenvironment (ROI A, vascular fraction of 1%), a vascularization degree comparable with published values for prostate cancers (ROI B, vascular fraction of 2.34%) and a highly vascularized prostate tumor microenvironment (ROI C, vascular fraction of 3.2%). The fraction of physoxia in the microenvironment goes from 8.9% in ROI A to 31.3% in ROI C showing a proportional behavior with respect to the vascular fraction. On the other hand, the fraction of tissue classified as radiobiological hypoxia is 36.3% in ROI C and increases up to 72.7% in ROI A, demonstrating an inverse correlation with the degree of vascularization. Regions of physiological hypoxia seem to be primarily dependent on the local vessel density, and inter-vessel distance.

### 3.2. Dose Distribution in Tumor Microenvironment

**Figure 3:**
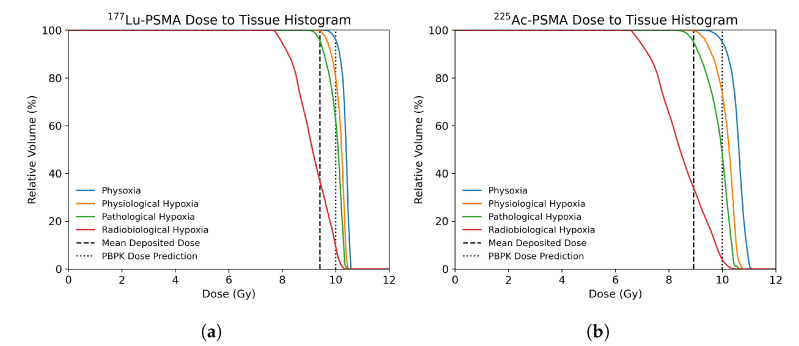

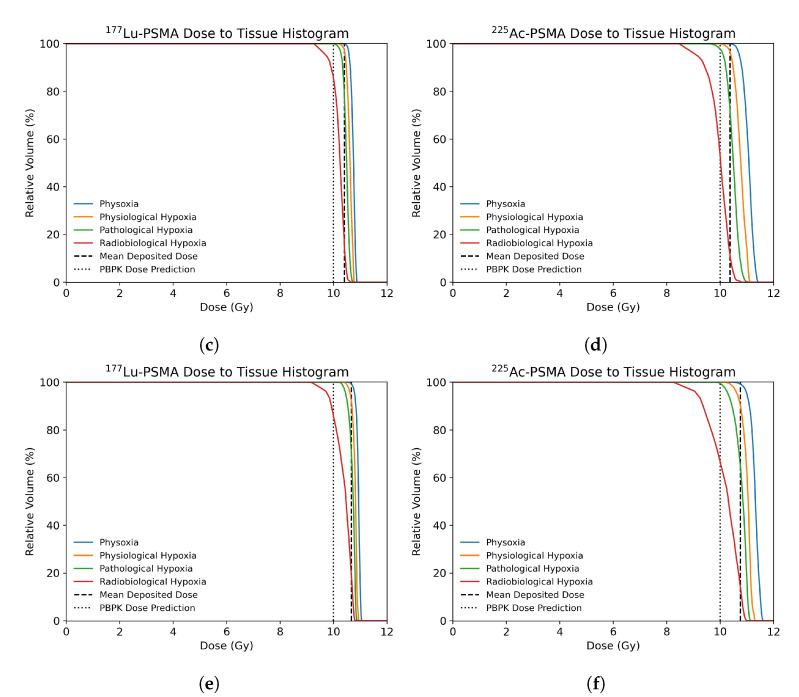
(**a**,**c**,**e**) Cumulative dose to tissue histogram of deposited dose in ROI A, ROI B and ROI C, respectively, within each tissue segment with ${}^{177}$Lu-PSMA treatment at twenty days post-injection. (**b**,**d**,**f**) Cumulative dose to tissue histogram of deposited dose in ROI A, ROI B and ROI C, respectively, within each tissue segment with ${}^{225}$Ac-PSMA treatment at twenty days post-injection.

Once the activity distribution was recovered, the Equation (9) was solved to calculate the dose distribution in the ROI. Figure 3 shows the cumulative dose to tissue histograms (DTH) calculated for the three ROIs and for each radionuclide at twenty days post injection. The DTH metric, similarly to the cumulative dose to volume histograms (DVH) commonly used in clinical practice, has been established in order to analyse the dose delivered to any defined tissue in the tumor microenvironment.

In all cases a more homogeneous dose distribution is produced by ${}^{177}$Lu. The higher homogeneity of the dose distribution is highlighted by the steep shoulders of the DTHs for all the considered tissues. This is due to the higher “cross-fire” induced by the higher range covered by the β particles with respect to the α particles emitted by ${}^{225}$Ac. Moreover, the DTH metric shows that in the poorly vascularized tissues (ROI A) only the 10% of radiobiological hypoxic tissue receives the target dose using ${}^{177}$Lu-PSMA-ligand treatment. This percentage drops down to 5% using ${}^{225}$Ac. In highly vascularized tissues (ROI C) the percentage of hypoxic tissue receiving the target dose increases to more than 85% and 65% for the ${}^{177}$Lu and ${}^{225}$Ac-PSMA-ligands, respectively.

**Figure 4:**
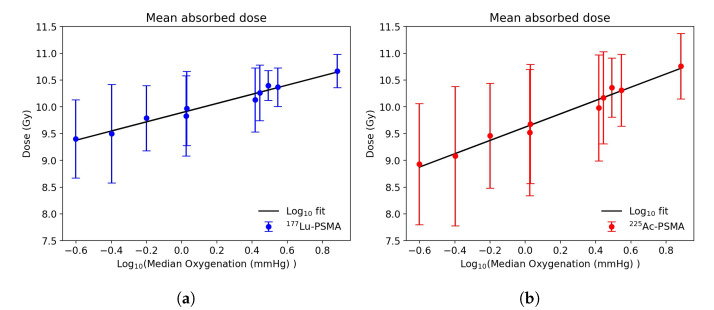
Relationship between the mean absorbed dose and the median oxygenation for ^177^Lu-PSMA treatment (**a**) and ^225^Ac-PSMA treatment (**b**). The vertical lines represent the dose standard deviation within each examined ROI.

The statistical analysis performed in all the ROIs shows an overall higher standard deviation in the dose distributions obtained with ${}^{225}$Ac with respect to ${}^{177}$Lu. A linear model was fitted to explore the relationship between the mean deposited dose and the $Log_{10}$ median tissue oxygenation (Figure 4). For both ${}^{177}$Lu-PSMA and ${}^{225}$Ac-PSMA a high coefficient of determination ($R^{2}=0.97$ and 0.98, respectively) was calculated, indicating a good model fit. A steeper slope was calculated in the case of ${}^{225}$Ac-PSMA with respect to ${}^{177}$Lu-PSMA (1.24 and 0.86, respectively).

### 3.3. Radiobiological Efficacy Analysis

**Figure 5:**
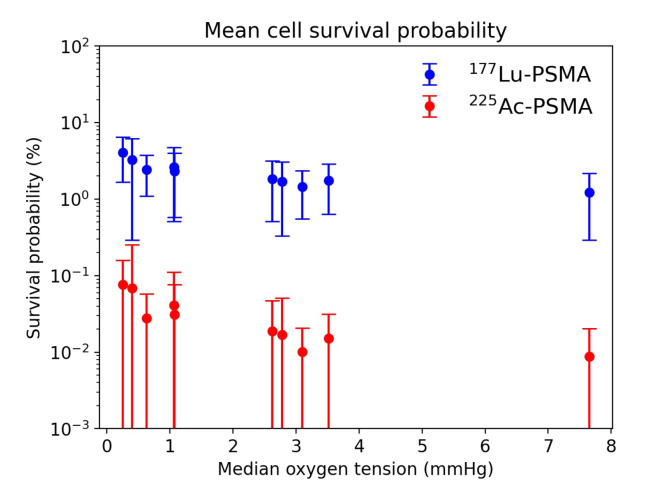
Cell survival probability depending on the ROI median oxygen tension for ${}^{177}$Lu-PSMA and ${}^{225}$Ac-PSMA treatments.

Applying the linear-quadratic model described in Section 2.7 to the dose maps, the cell survival probability for both ${}^{225}$Ac-PSMA-ligands and ${}^{177}$Lu-PSMA-ligand treatments has been calculated. The statistical analysis performed in all the ROIs shows an overall higher survival probability of tumor cells when the radiopharmaceutical is labelled with ${}^{177}$Lu instead of ${}^{225}$Ac (Figure 5). While the mean irradiation dose is comparable for both radiopharmaceuticals, the ${}^{225}$Ac-PSMA-ligand has a overall significantly higher cell-killing potency. This finding is consistent with the reported higher probability of double-stranded break induction by the α particles, which are more difficult for the cell to repair.

**Figure 6:**
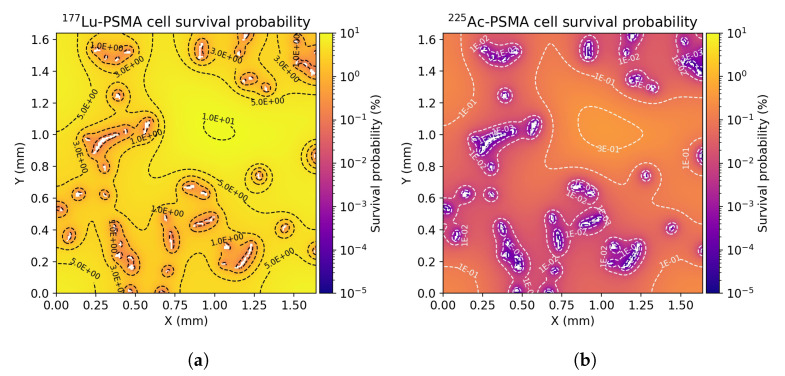

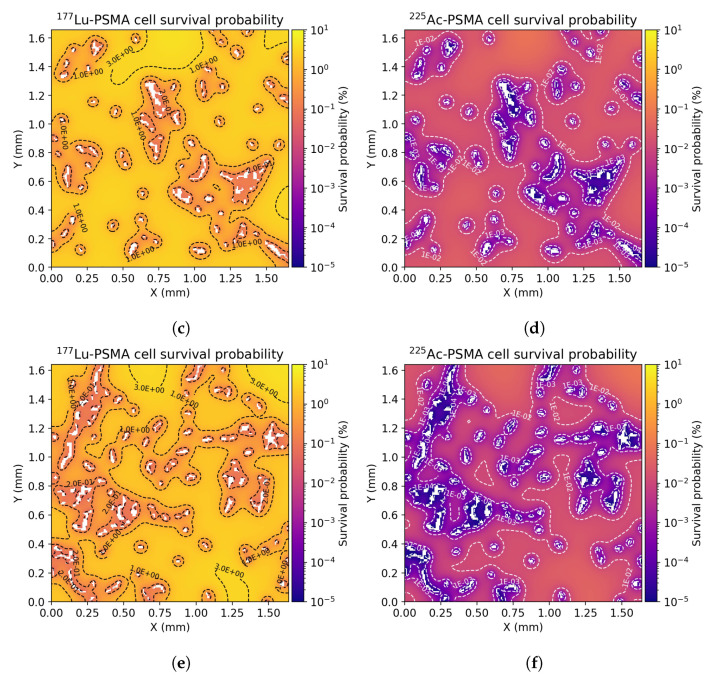
(**a**,**c**,**e**) 2D distribution of cell survival probabilities in ROI A, ROI B and ROI C, respectively, within each tissue segment with ${}^{177}$Lu-PSMA treatment at twenty days post-injection. (**b**,**d**,**f**) 2D distribution of cell survival probabilities in ROI A, ROI B and ROI C, respectively, within each tissue segment with ${}^{225}$Ac-PSMA treatment at twenty days post-injection.

Figure 6 depicts the cell survival probability distribution for the examined ROIs. The cell survival probability can be four order of magnitude higher for ${}^{177}$Lu with respect to ${}^{225}$Ac in vessels neighborhood. This difference can decrease by two orders of magnitude at large distances from vessels. While the ${}^{177}$Lu-PSMA-ligand is overall less lethal, the longer range of β particles enables the ${}^{177}$Lu based treatment to be less dependent on the degree of domain vascularization. This can be also seen by the smaller standard deviation in the mean cell survival probability for all the examined ROIs.

## 4. Discussion

In this work the influence of tumor hypoxia on the treatment outcome for PSMA-directed RLT with ${}^{177}$Lu and ${}^{225}$Ac has been investigated. The established hybrid histology-driven in silico model allowed to describe the dynamics of PSMA-ligands, the dose distribution and the radio-biological efficacy at the tumor microenvironment scale. The platform presented in this work is composed of four main models: a tissue oxygenation model based on the solution of a reaction-diffusion partial differential equation, a three compartment convection–reaction–diffusion model to simulate the propagation of radiopharmaceuticals in the tumor microenvironment, a kernel-based method for the dose calculation and a linear-quadratic model used to estimate the cell survival probability depending on the type of particle and the region oxygenation. The model parameters were derived from the existing literature and a validated PBPK model has been adopted to calculate the AIF.

In tumors, hypoxia occurs in regions with low vascular density [53]. On the contrary to the existing literature, our findings suggest that the dose distribution for both α and β emitters in poorly vascularized domains is heterogeneous. The low vascularization and the limited penetration of the radiopharmaceutical into the tumor microenvironment due to the high affinity of PSMA-ligands to the receptors, result in an overall low mean dose of radiation and a very low percentage of hypoxic tissue receiving the prescribed dose. Indeed, for the same mean irradiation dose 20 days p.i., the DTH metric shows that in poorly vascularized ROIs, the dose delivered to the radiobiological hypoxic tissues is suboptimal for both ${}^{177}$Lu-PSMA-ligands and ${}^{225}$Ac-PSMA-ligands. While the range of the β particles is high compared to the size of hypoxic regions, the “cross-fire” effect is not sufficient to completely homogenize the dose distribution within tissues. This could have an important effect on the treatment efficacy. On the other hand, a more homogeneous PSMA-ligands distribution is achieved in highly vascularized domains leading to a higher mean dose and a homogeneous dose distribution for both types of radiopharmaceuticals. In these ROIs, the percentage of radiobiological hypoxic tissue receiving the target dose is more than 85% and 65% for the ${}^{177}$Lu and ${}^{225}$Ac-PSMA-ligands, respectively.

Tumor hypoxia is also considered a critical factor associated with the resistance to conventional radiotherapy [4], where the X-ray-induced free radicals lead to DNA damage in a manner that is strongly dependent on the tissue oxygenation. While the lack of oxygen is less impactful on the treatment outcome compared to therapies with X- or γ-ray, for the emitted α and β particles the effect still exists. This, in combination with a lower deposited dose due to a limited penetration of the radiopharmaceutical and a low vascular density, leads to a significantly lower cell killing potency and hence treatment efficacy. Our findings suggests that for both α and β emitters, to a higher degree of oxygenation of the ROIs corresponds a lower cell survival probability. The previous findings on the significantly higher efficacy of the ${}^{225}$Ac-PSMA-ligands therapy are confirmed. This is consistent with the higher probability of DSB induction by the α particles. However, our model suggests a lower dependency of the treatment outcome on the median oxygenation of the tissue for the treatment delivered with ${}^{177}$Lu with respect to ${}^{225}$Ac as shown in Figure 5.

This model is subject to some limitations. Two main types of hypoxia can be defined: Acute and chronic hypoxia. While chronic hypoxia is a quasi-steady state condition, acute hypoxia occurs due to fluctuations in red cell flux or hemoglobin saturation over minutes to hours [54,55]. In this study the morphological organization of the microvessels and the vessel wall permeability to oxygen ($L_{O_{2}}$) and PSMA-ligands ($L_{v}$) was modelled as being constant. Therefore, as for previous theoretical studies, only chronic hypoxia was modeled [23,24,26,27,28,29]. While the perfusion of oxygen and pharmaceuticals can be highly heterogeneous especially in tumors [34], chronic hypoxia is the dominant type in tumor microenvironment (>70% [56]). The levels of median pO${}_{2}$ calculated with our model in each ROI are in agreement with the existing in silico and in-vivo published studies [40] suggesting that the results of this work may still be meaningful. The vessel map generation could neglect some small vessels. This could bring to an inaccurate estimation of oxygen or radiopharmaceuticals supply. However, the visual comparison performed for all the ROIs confirmed the very good accuracy of the method. Moreover, the time activity curves (TACs) calculated in the ROIs with the PSMA-ligands dynamics models share the same characteristics of the PBPK-derived TACs [38]. This suggests that the model developed in this work is consistent with its macroscopical approximation giving at the same time a highly detailed description at a microscopical scale. Another source of inaccuracy is the use of the two-dimensional approximation. This limits the diffusion of the molecules only radially and neglects the contribution of the molecules moving in and out of the simulation plane [27]. Moreover, the vessel maps do not take into account vessels that are nearby but outside its thickness. In the actual tissue, these vessels can supply drugs and oxygen to the ROI. Therefore, the use of a 2D model systematically overestimates distances from tissue points to the nearest vessel, which could affect the predicted distribution of solutes. Nevertheless, the overall 3D inflow and outflow effect may be cancelled and the 2D based simulation was found to match the experimental results without large errors [29]. The same limitation can be referred to in the calculation of the dose distribution. The multi-compartment approach is very common in pharmacokinetic and in tumor microenvironment modelling where binding and internalization processes have to be described [57,58,59]. However, the assumptions of homogeneous cell density, of systematic division of the simulation space into sub-compartments and of constant diffusion coefficients are affected by the high tumour heterogeneity. A more realistic alternative would consist of a more detailed morphological segmentation of the tumour microenvironment. The S-values calculated for kernel construction were based on MIRD-cell (V2.0) that assumes uniform cell size and intercellular distance as well as spherical cell and nucleus geometry, which are all factors that have been shown to influence the deposited dose. The overall contribution of each of these factors to the dose accuracy is typically small [48]. However, deviations might occur for cells whose diameter substantially differs from the used value. Dose overestimation is also plausible in regions with small cellular density as the kernel assumes that each pixel in the domain is populated by a predefined fraction of a cell.

Radiation therapy has been linked with vascular remodeling [60]. In this model the vessel map derived from staining represents a “snapshot” of the tumor at a given time. For this reason, the dynamics of abnormal vessel growth that arise in tumor tissue due to angiogenesis [61] are not taken into account. Furthermore, it has been shown that radiation therapy can cause hypoxia [62] or upregulate HIF-1 expression [63], which in turn leads to a development and selection of more resistant cells [64,65]. Moreover, the biological effects induced by other pharmaceuticals, e.g., the effect of androgen-deprivation therapy with bicalutamide in selecting more resistant cells to the treatment [66], are not considered at this stage. We intend to consider these factors as potential model development.

The theoretical predictions of this paper should be further investigated experimentally. Autoradiography has been used in RLT research to characterize the radiopharmaceutical distribution in salivary glands [67,68]. Additionally, animals can be scanned with dynamic PET to obtain individual AIF for the simulation. The simulation results with the individual AIF can be compared with the obtained autoradiography images. The verification of the radiobiological effects will be performed by following the treatment effects in different groups of animals transplanted with the same type of tumor.

## 5. Conclusions

In this paper has been presented a hybrid in silico model to study the role of chronic hypoxia on the treatment outcome for PSMA-directed RLT. The incorporation of histology-derived microvascular networks in PBPK-integrated convection-reaction-diffusion model allows the investigation of the microdosimetry of ${}^{177}$Lu and ${}^{225}$Ac PSMA-directed radioligand therapy in the heterogeneous tumor microenvironment.

Although the higher homogeneity of the dose distribution delivered by ${}^{177}$Lu with respect to ${}^{225}$Ac is confirmed, the DTH metric shows that in poorly vascularized ROIs, i.e. hypoxic areas, the dose delivered to the radiobiological hypoxic tissues can be suboptimal for both ${}^{177}$Lu-PSMA-ligands and ${}^{225}$Ac-PSMA-ligands. Nevertheless, for the same mean irradiation dose 20 days p.i., the ${}^{225}$Ac-PSMA-ligand has an overall significantly higher cell-killing potency. The development of advanced in silico modeling may assist the personalization of RLT.

## Figures and Tables

**Table 1:** Model parameter values used in PSMA-ligands dynamics simulations.

Symbol	Parameter	Value	Reference
$L_{v}$	Vessel wall permeability	$3.3\times 10^{-4}$ cm s${}^{-1}$	[37]
$D_{\mathrm{PSMA}}$	Diffusivity	$8.7\times 10^{-7}$ cm${}^{2}$ s${}^{-1}$	[36]
$R_{f}$	Molecule/Carrier movement coefficient	1	[36]
$R_{0}$	Receptor density	$4.089\times 10^{-2}$ nmol ml${}^{-1}$	fitted
$k_{\mathrm{on}}$	Association rate	$7.7\times 10^{-1}$ ml nmol${}^{-1}$ s${}^{-1}$	[38]
$k_{\mathrm{off}}$	Dissociation rate	$7.7\times 10^{-4}$ s${}^{-1}$	[38]
$k_{\mathrm{int}}$	Internalization rate	$1.67\times 10^{-5}$ s${}^{-1}$	[38]
$k_{\mathrm{rel}}$	Release rate	$2.67\times 10^{-6}$ s${}^{-1}$	[38]
$FV_{i}$	Fractional interstitial volume	39%	[38]
$FV_{c}$	Fractional cellular volume	61%	[38]
$\lambda_{\mathrm{dec}}$	${}^{177}$Lu decay constant	$1.197\times 10^{-6}$ s${}^{-1}$	
$\lambda_{\mathrm{dec}}$	${}^{225}$Ac decay constant	$8.087\times 10^{-7}$ s${}^{-1}$	

**Table 2:** Model parameter values used in tissue oxygenation simulations.

Symbol	Parameter	Value	Reference
$L_{O_{2}}$	Vessel wall permeability to O${}_{2}$	$4.1\times 10^{-2}$ cm s${}^{-1}$	[27,29]
$D_{O_{2}}$	Oxygen diffusivity	$2.0\times 10^{-5}$ cm${}^{2}$ s${}^{-1}$	[27,29]
$P_{\mathrm{ie}}$	Intraerythrocyte pO${}_{2}$ in tumors	40 mmHg	[27,29]
$M_{0}$	Maximum O${}_{2}$ consumption rate	15 mmHg s${}^{-1}$	[27,29]
$P_{0}$	Michaelis-Menten coefficient of oxygen consumption	2.0 mmHg	[27,29]

**Table 3:** Parameters used for the cells survival probability calculations.

Parameter	Physoxia	Radiobiological Hypoxia	Ref.
α	0.15 Gy${}^{-1}$	0.107 Gy${}^{-1}$	[51]
β	0.048 Gy${}^{-2}$	0.024 Gy${}^{-2}$	[51]
D${}_{0}$	0.7 Gy	1.18 Gy	[52]

## Data Availability

The Python script used to generate ROIs can be downloaded at https://www.dropbox.com/sh/fy5ud6f7vovh4ty/AABPbDhbONdqus4OAJEFNUmGa?dl=0, accessed date 13 June 2021.
